# Biocontrol mechanism by root-associated *Bacillus amyloliquefaciens* FZB42 – a review

**DOI:** 10.3389/fmicb.2015.00780

**Published:** 2015-07-28

**Authors:** Soumitra Paul Chowdhury, Anton Hartmann, XueWen Gao, Rainer Borriss

**Affiliations:** ^1^Helmholtz Zentrum München – German Research Center for Environmental Health (GmbH), Research Unit Microbe–Plant InteractionsNeuherberg, Germany; ^2^College of Plant Protection, Nanjing Agricultural UniversityNanjing, China; ^3^Key Laboratory of Monitoring and Management of Crop Disease and Pest Insects, Ministry of AgricultureNanjing, China; ^4^ABiTEP GmbHBerlin, Germany; ^5^Fachgebiet Phytomedizin, Institut für Agrar- und Gartenbauwissenschaften, Humboldt-Universität zu BerlinBerlin, Germany

**Keywords:** *Bacillus amyloliquefaciens plantarum*, FZB42, induced systemic resistance (ISR), non-ribosomal synthesized lipopeptides (NRPS), non-ribosomal synthesized polyketides (PKS), volatiles, plant growth promoting bacteria (PGPR)

## Abstract

*Bacillus amyloliquefaciens* subsp. *plantarum* FZB42 is a Gram-positive model bacterium for unraveling plant–microbe interactions in Bacilli. In addition, FZB42 is used commercially as biofertilizer and biocontrol agent in agriculture. Genome analysis of FZB42 revealed that nearly 10% of the FZB42 genome is devoted to synthesizing antimicrobial metabolites and their corresponding immunity genes. However, recent investigations *in planta* demonstrated that – except surfactin – the amount of such compounds found in vicinity of plant roots is relatively low, making doubtful a direct function in suppressing competing microflora including plant pathogens. These metabolites have been also suspected to induce changes within the rhizosphere microbial community, which might affect environment and plant health. However, sequence analysis of rhizosphere samples revealed only marginal changes in the root microbiome, suggesting that secondary metabolites are not the key factor in protecting plants from pathogenic microorganisms. On the other hand, adding FZB42 to plants compensate, at least in part, changes in the community structure caused by the pathogen, indicating an interesting mechanism of plant protection by beneficial Bacilli. Sub-lethal concentrations of cyclic lipopeptides and volatiles produced by plant-associated Bacilli trigger pathways of induced systemic resistance (ISR), which protect plants against attacks of pathogenic microbes, viruses, and nematodes. Stimulation of ISR by bacterial metabolites is likely the main mechanism responsible for biocontrol action of FZB42.

## Introduction

Plant rhizosphere is a highly competitive environment in which micro-organisms are abundantly present due to the availability of nutrients actively secreted by the plant root and mucilage. Some of these bacteria which are living within or in the vicinity of plant roots and supporting plant growth are generally referred as being plant-growth-promoting rhizobacteria (PGPR; Kloepper et al., 1980). In many cases their plant growth promoting activity is linked with their ability to suppress soil-borne plant pathogens (bacteria and microfungi), occurring in the competing microflora. Different mechanisms are discussed in this context. Besides production of antimicrobial (“antibiotics”), antiviral and nematicidal compounds, also stimulation of plant induced systemic resistance (ISR; Doornbos et al., 2012), and a beneficial effect on the composition of the host-plant microbiome might contribute to their suppressive effect (Erlacher et al., 2014).

The aim of the present review is to describe the “state of the art” in elucidating interactions within the tripartite system consisting of beneficial bacterium, the pathogen and the plant by using *Bacillus amyloliquefaciens* FZB42 as a model. The aerobic-endospore-forming rhizobacteria belonging to *B. amyloliquefaciens* subsp. *plantarum* (Borriss et al., 2011) are known for enhancing yield of crop plants and for their biocontrol function directed against plant pathogens. The type strain of the subspecies, FZB42^T^, is commercially used as biocontrol bacterium being especially efficient against fungal and bacterial pathogens (Borriss, 2011). Its plant colonizing ability was demonstrated with a GFP-labeled FZB42 derivative on *Lemna minor, Arabidopsis thaliana*, maize, tomato, and lettuce using confocal laser scanning microscopy (Fan et al., 2011, 2012). Beneficial effects on plant growth and disease suppression were documented for *B. amyloliquefaciens* FZB42 on tomato, cucumber, cotton, tobacco, and lettuce for example (Grosch et al., 1999; Yao et al., 2006; Guel et al., 2008; Wang et al., 2009; Chowdhury et al., 2013, 2015).

**FIGURE 1:**
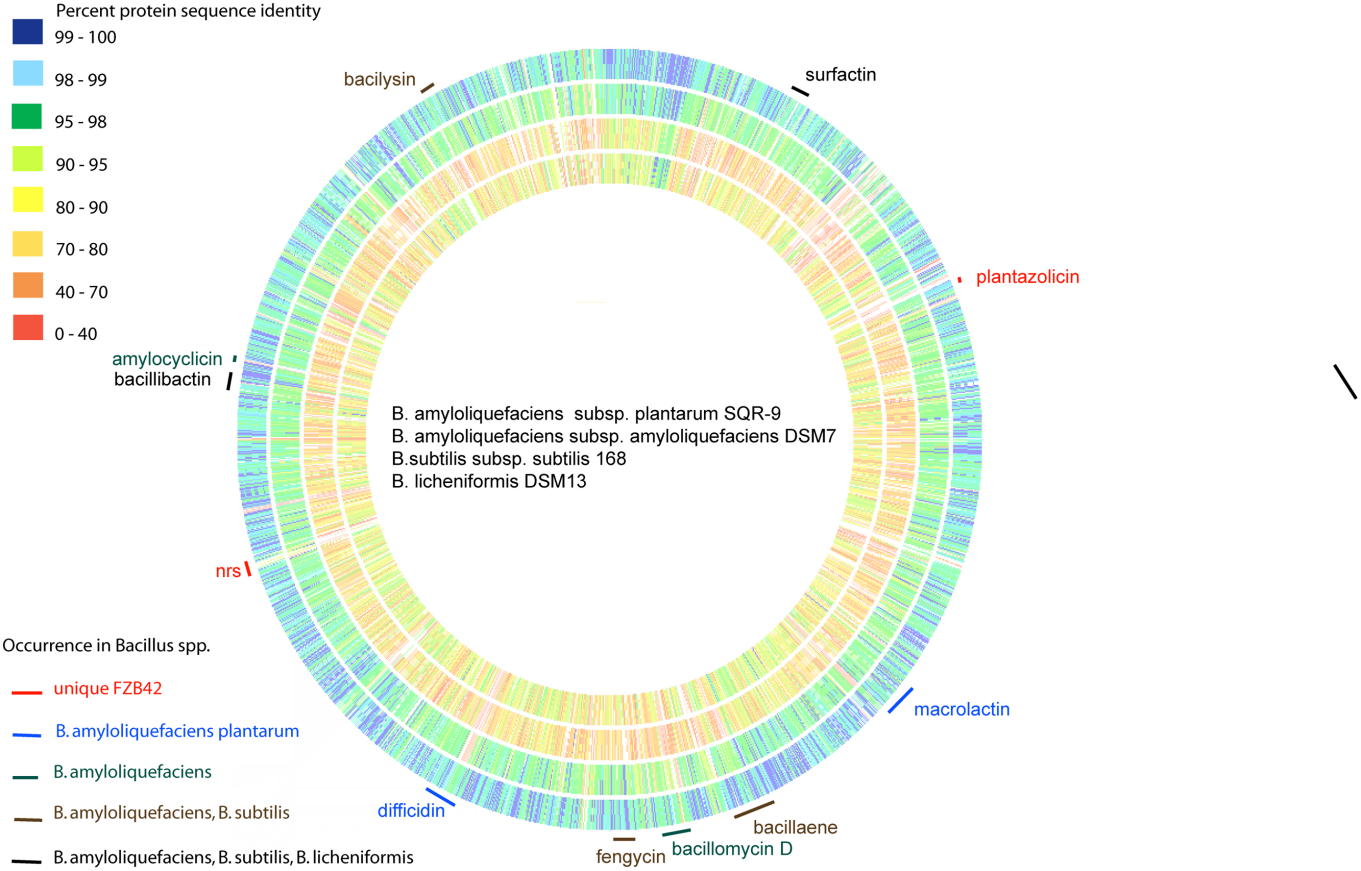
**Genome comparison of the type strains of *Bacillus amyloliquefaciens, B. subtilis*, and *B. licheniformis*.** The whole genomes of *B. amyloliquefaciens plantarum* SQR-9 (outside circle), *B. amyloliquefaciens amyloliquefaciens* DSM7^T^ (second circle), *B. subtilis subtilis* 168^T^ (third circle), and *B. licheniformis* DSM13^T^ (inner circle) were aligned with FZB42^T^ using the RAST server (Aziz et al., 2008). The color code indicates % similarity of single gene products. The gene clusters responsible for non-ribosomal synthesis of the polyketides macrolactin and difficidin are unique in *B. amyloliquefaciens* subsp. *plantarum*. The iturin gene cluster (bacillomycin D) occurs also *in B. amyloliquefaciens* subsp. *amyloliquefaciens*.

A comparison of the genomic sequence of FZB42 with that of the non-plant-associated *B. amyloliquefaciens* type strain DSM7^T^ revealed significant differences in the genomic sequences of both strains (Rueckert et al., 2011). The strains have in common 3345 CDS residing in their core genomes; whilst 547 and 344 CDS were found to be unique in FZB42^T^ and DSM7^T^, respectively. Notably, ability to synthesize non-ribosomally the antibacterial polyketides macrolactin and difficidin is an unique feature of the subspecies *plantarum*, whilst capability to synthesize an iturin-like antifungal lipopeptide (LP) is shared with subsp. *amyloliquefaciens* but not with the other members of the *B. subtilis* species complex (**Figure 1**).

In addition to FZB42^T^, the genomes of other *B. amyloliquefaciens plantarum* strains have become recently available (Borriss, 2013). The core-genome formed by 15 *B. amyloliquefaciens plantarum* genomes contains 3,151 genes, the pan-genome more than 6,000 genes, suggesting a high degree of flexibility in the genomes of such plant-associated *B. amyloliquefaciens* strains. Fifty-four genes were identified as being unique for subspecies *plantarum* and did not occur in the free-living soil bacterium *B. amyloliquefaciens* subsp. *amyloliquefaciens*, for e.g., gene clusters involved in synthesis of polyketides, and in carbon metabolism (Qiao et al., 2014).

## Secondary Metabolites with Biocontrol Function

Analysis of the whole FZB42 genome revealed an impressive capability to produce a diverse spectrum of different secondary metabolites aimed to suppress harmful microbes and nematodes living within the plant rhizosphere (Chen et al., 2007). In total, 11 gene clusters (**Table 1**) representing more than 9% of the genome are devoted to synthesizing antimicrobial metabolites (Chen et al., 2009a; Borriss, 2013). By contrast, the genomes of the closely related non-plant associated members of the *B. subtilis* species complex devote only around 5% of their capacity in synthesis of antimicrobials.

**Table 1:** Genes and gene cluster encoding for biocontrol metabolites in *Bacillus amyloliquefaciens plantarum* FZB42.

Metabolite	Genes and gene cluster	Size	Function	Expression *in situ*	Effect against
**Sfp-dependent non-ribosomal synthesis of lipopeptides**					
Surfactin	*srfABCD*	32.0 kb	Biofilm, ISR	Strong, during root colonization	Virus
Bacillomycin D	*bmyCBAD*	39.7 kb	Direct suppression, ISR	Weak, during root colonization	Vungi
Fengycin	*fenABCDE*	38.2 kb	Direct suppression, ISR	Weak, during root colonization	Fungi
Bacillibactin	*dhbABCDEF*	12.8 kb	Siderophore	During iron deficiency in soil	Microbial competitors
Unknown	*nrsABCDEF*	17.5 kb	Unknown	Unknown	Unknown
**Sfp-dependent non-ribosomal synthesis of polyketides**					
Macrolactin	*mlnABCDEFGHI*	53.9 kb	Direct suppression	Not shown	Bacteria
Bacillaene	*baeBCDE,acpK, baeGHIJLMNRS*	74.3 kb	Direct suppression	Not shown	Bacteria
Difficidin	*dfnAYXBCDEFGHIJKLM*	71.1 kb	Direct suppression	Not shown	Bacteria
**Sfp-independent non-ribosomal synthesis**					
Bacilysin	*bacABCDE,ywfG*	6.9 kb	Direct suppression	Not shown	Bacteria, cyanobacteria
**Ribosomal synthesis of processed and modified peptides (bacteriocins)**					
Plantazolicin	*pznFKGHIAJC DBEL*	9.96 kb	Direct suppression	Unknown	*B. anthrax*, nematodes
Amylocyclicin	*acnBACDEF*	4.49 kb	Direct suppression	Unknown	Closely related bacteria
**Synthesis of volatiles**					
Acetoin/2,3-butandiol	*bdh,alsDRS*	3.6 kb	ISR	During root colonization	Plant pathogens

According to numerous *in vitro* studies it is widely assumed that its antifungal activity is due to non-ribosomal synthesis of the cyclic LP bacillomycin D and fengycin (Koumoutsi et al., 2004), whilst its antibacterial activity is mainly due to non-ribosomally synthesized polyketides (Chen et al., 2006), and bacilysin (Chen et al., 2009b), and ribosomally synthesized bacteriocins (Scholz et al., 2011, 2014).

## Lipopeptides Direct Antifungal Activity

**FIGURE 2:**
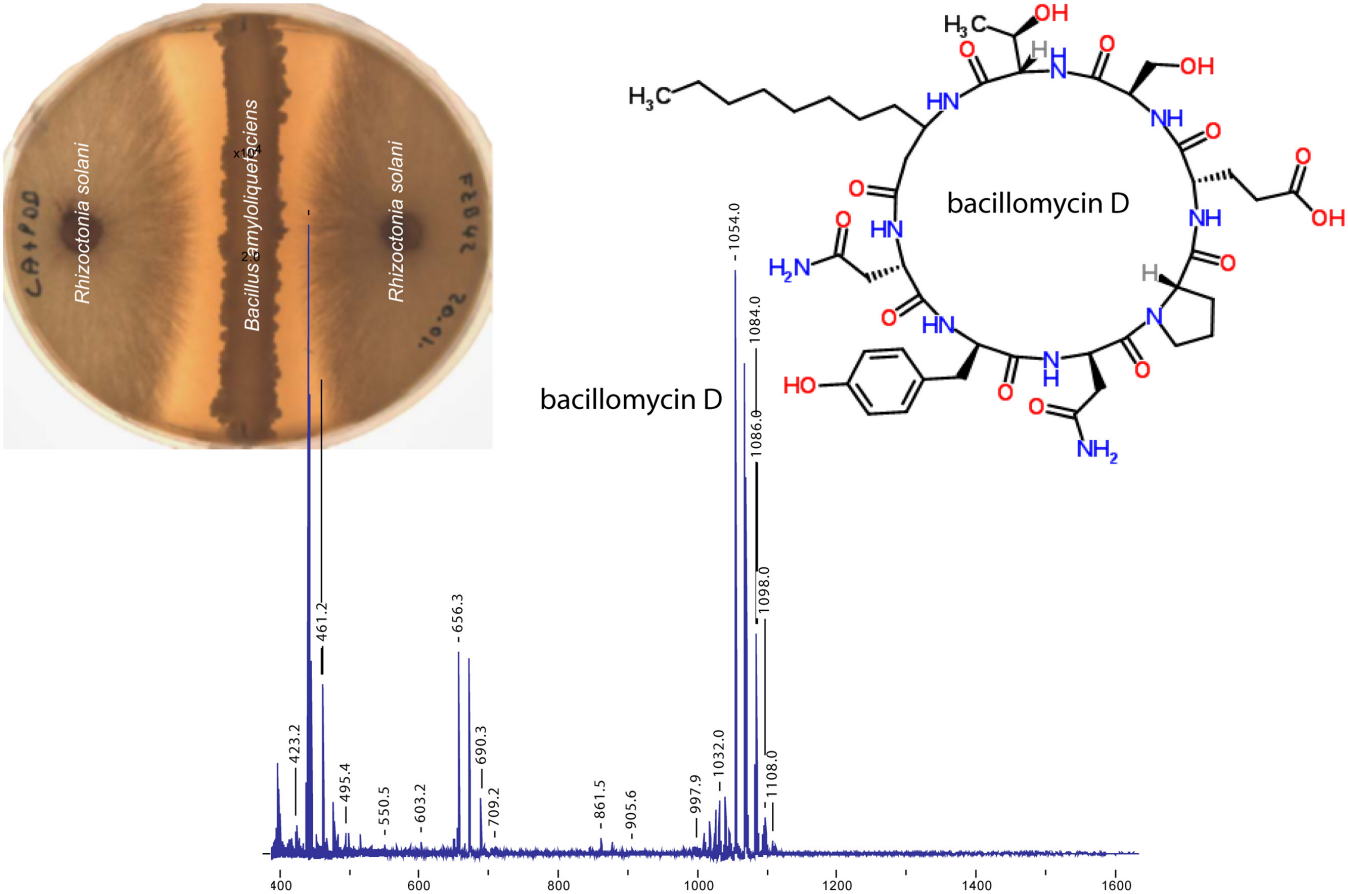
**Effect of FZB42 on *Rhizoctonia solani*.** A clear inhibition zone indicating growth suppression of the fungal pathogen is visible on agar plates simultaneously inoculated with both microbes. Bacillomycin D was detected as the only prominent compound by Matrix-Assisted Laser Desorption/Ionization coupled to time of flight (MALDI TOF) mass spectrometry of samples taken from the surface of the agar plate within the inhibition zone (compiled from data obtained by J. Vater, TUB and K. Dietel, ABiTEP GmbH).

Lipopeptides are non-ribosomally synthesized by peptide synthetases (NRPS). NRPS are giant enzymes composed of modules that house repeated sets of functional domains, which select, activate, and couple amino acids drawn from a pool of nearly 500 potential building blocks (Walsh et al., 2013). Five gene cluster involved in non-ribosomal synthesis of cyclic LP and the iron-siderophore bacillibactin were identified in the genome of FZB42 (**Table 1**). Three of the respective gene clusters were assigned for synthesis of surfactin, fengycin, and bacillomycin D. Bacillomycin D was identified as being the most powerful antifungal metabolite *in vitro* produced by FZB42 (**Figure 2**). The heptapeptide moiety of bacillomycin D, belonging to the iturin family of cyclic LP, is attached to a β-amino fatty acid chain of variable length (C_14_–C_17_). The peptide moiety of the heptapeptide surfactin is linked to a β-hydroxyl fatty acid (C_12_–C_16_), whilst the fengycin decapeptides are linked to a β–hydroxyl fatty acid chain (C _14_–C_18_). Their synthesis is accomplished by multimodular peptide synthetases and depends on a functional phospho-pantheinyl transferase (Sfp) which transfers 4′- phosphopantetheine from coenzyme A to the carrier proteins during non-ribosomal synthesis.

For a long time the plant protective activity of PGPR has been correlated with the potential to secrete a wide array of antibiotic compounds upon growth as planktonic cells in isolated cultures under laboratory conditions (Velivelli et al., 2014). A recent comparative study performed with six strains belonging to the *B. subtilis/amyloliquefaciens* species complex corroborated our earlier finding that antifungal activity is linked with the ability to produce cyclic LP. Remarkably, production of iturin and fengycin in *B. amyloliquefaciens* was enhanced in presence of certain phytopathogens (Cawoy et al., 2015). This is in line with a recent finding that non-ribosomal synthesis of antifungal and antibacterial compounds including bacillibactin is stimulated in presence of plant pathogens under laboratory conditions (Li et al., 2014).

We determined expression of the corresponding secondary metabolites by Matrix-Assisted Laser Desorption/Ionization coupled to time of flight (MALDI TOF) mass spectrometry from FZB42 cultures grown in liquid Landy medium under laboratory conditions. Except the orphan *nrs* gene cluster, all expected bioactive compounds were synthesized in reasonable amounts. However, the iron siderophore bacillibactin was detected only under iron-deprived conditions. In recent years, it has become doubtful, that synthesis of metabolites by the planktonic cells grown under laboratory conditions does correspond to their capability to produce those compounds under true environmental conditions, e.g., when grown in biofilm-related structures on the surface of plant tissues.

During last years, Ongena et al. (2007) performed pioneering work in elucidating antibiotic production *in planta* using MALDI Mass Spectrometry Imaging (MSI). They investigated antibiotic production in a gnotobiotic system in which the plantlet and the associated *B. amyloliquefaciens* S499, a close relative of FZB42, were growing on a gelified medium covering the MALDI target plate. Under these conditions S499 grows as biofilm on the surface of the plant roots, allowing exact assays of secondary metabolites in the vicinity of root surface. Surfactins were detected in the root environment in much higher relative amounts, which are representing more than 90% of the whole LP production, and their synthesis is rapidly progressing during early biofilm formation. Syntheses of iturin and fengycin were also detected, but found delayed until the end of the aggressive phase of colonization (Nihorimbere et al., 2012; Debois et al., 2014). Earlier experiments performed with FZB42 colonizing duckweed (*Lemna minor*) plantlets corroborated that surfactin is the most prominent compound which could be detected by MALDI TOF MS in the plant–bacteria system (Idris et al., 2007). Recently, we examined the *in situ* production of selected secondary metabolites by FZB42 in the lettuce rhizosphere using ultra-high performance liquid chromatography coupled to time of flight mass spectrometry (UHPLC-qToF-MS). The LP surfactin, fengycin and bacillomycin D were identified in the rhizosphere of lettuce plants grown in an axenic system and bacterized with FZB42 for 7 days. Interestingly, the presence of the phytopathogen *Rhizoctonia solani* in the axenic system increased the production of surfactin and bacillomycin D by FZB42 (Chowdhury et al., 2015). The increase in the production of bacillomycin D in the presence of *R. solani* pointed to an antibiosis effect and the recognition and response of FZB42 to the fungal stimulus. This was in accordance with the recent investigation with different strains of *B. amyloliquefaciens* including FZB42 showing enhanced production of iturins and fengycins in response to signals emitted by phytopathogens like *Fusarium oxysporum* and *Botrytis cinerea* (Cawoy et al., 2015).

An early surfactin secretion could be of biological relevance since this LP, although less fungitoxic than iturins and fengycins, is essential for moving on tissues (Kinsinger et al., 2003) and for matrix formation in biofilms (Hofemeister et al., 2004; Lopez et al., 2009a,b). Considering the relative low amounts of the fungitoxic iturins and fengycins in vicinity of plant roots, it can be concluded that their biocontrol effect is possibly less important. The same is true for the iron siderophore bacillibactin, which was not detected, possibly due to a relative high level of accessible iron ions under the conditions of the artificial plant–bacteria associations applied in these studies.

## Non-Ribosomal Polyketides and Bacilysin Direct Antibacterial Activity *In Vitro*

The polyketides, non-ribosomally synthesized by FZB42 (Chen et al., 2006; Schneider et al., 2007), have been extensively reviewed previously (Chen et al., 2009a,c; Borriss, 2013). The three gene clusters encoding the modularly organized polyketide synthases (PKS) for the synthesis of bacillaene, macrolactin, and difficidin cover nearly 200 kb, and are the largest ones, which are occurring in the FZB42 genome (**Table 1**). Difficidin is the most effective antibacterial compound produced by FZB42^T^, but also macrolactin and bacillaene possess antibacterial activity. Difficidin is efficient in suppressing plant pathogenic bacterium *Erwinia amylovora*, which causes fire blight disease in orchard trees (Chen et al., 2009b).

Another product of non-ribosomal synthesis, the dipeptide bacilysin consisting of anticapsin and alanine moieties, was found as also being involved in suppression of *E. amylovora*. By contrast to the LP and polyketides mentioned above, bacilysin synthesis is not dependent on the Sfp PP-transferase. A mutant strain CH3, with a disruption of the *sfp* gene and unable to produce any polyketide or LP, was still able to synthesize bacilysin and to suppress *E. amylovora* (Chen et al., 2009b). Recent experiments, performed by the group of XueWen Gao, Nanjing Agriculture University, demonstrated that bacilysin, is efficient in suppressing *Microcystis aeruginosa*, the main causative agent of cyanobacterial bloom in lakes and rivers (Wu et al., 2014). However, corroborating these results in field trials has to be done. It is interesting to note that non-ribosomal LP such as the plant immunity elicitor surfactin or the highly fungitoxic iturins and fengycins were readily produced albeit in different time frames and quantities in the vicinity of plant roots, whilst polyketides, bacilysin, and other bioactive compounds have not been detected till now in plants colonized by *B. amyloliquefaciens* (Debois et al., 2014).

In light of these findings the question arises what is the physiological function of bacillaene and other polyketides, when these compounds are apparently not involved in inhibition of competitors in natural habitat? It has been suggested that sublethal concentrations of antibiotics may have a role as signaling molecule, e.g., in modulating transcription (Fajardo and Martinez, 2008). Interestingly, the polyketide bacillaene, produced in *B. subtilis* NCIB3610, functions as a significant defense protecting *Bacillus* cells from predation by *Myxococcus xanthus* (Müller et al., 2014).

## Bacteriocins Suppress Phytopathogenic Bacteria and Nematodes

Antimicrobial peptides, ribosomally synthesized as linear precursor peptides, remained unknown in *B. amyloliquefaciens* subsp. *plantarum* for a long time with one remarkable exception: mersacidin, a B-type lantibiotic, was detected in *Bacillus* sp. HIL Y85 (Chatterjee et al., 1992). The strain HIL Y85 was later classified as being *B. amyloliquefaciens plantarum* (Herzner et al., 2011). Nowadays, mersacidin production was also detected in *B. amyloliquefaciens* B9601-Y2 (He et al., 2012). Genes involved in mersacidin self-protection reside also in the genome of FZB42. Transfer of mersacidin biosynthesis genes from HIL Y85 resulted in efficient mersacidin production by the surrogate strain constructed from the FZB42 host (Herzner et al., 2011).

Another representative of the type B lantibiotics, amylolysin from *B. amyloliquefaciens* GA1, was recently described. These lantibiotics are active on an array of Gram-positive bacteria, including *Listeria* sp. and methicillin resistant *S. aureus* by interacting with the membrane lipid II (Arguelles Arias et al., 2013).

Driving force in our search for ribosomally synthesized peptides in FZB42 was the finding that the FZB42 mutant RS06, which is deficient in the Sfp-dependent synthesis of LP, polyketides, and in the Sfp-independent bacilysin production (Chen et al., 2009b), still produced an antibacterial substance active against *B. subtilis* HB0042. In fact, a metabolite (cpd1335) with a molecular mass of [M+H]^+^ = 1336 Da was assigned by MALDI TOF MS in FZB42 and in RS06, as well. The compound was named plantazolicin, PZN, and the respective gene cluster consisting of 12 genes was identified by cassette mutagenesis. Plantazolicin was characterized as a highly modified peptide undergoing several steps of modification after synthesis. It ruled out that it is a thiazole/oxazole-modified microcin (TOMM) resembling microcin B17 and streptolysin S. Plantazolicin displayed antibacterial activity toward closely related Gram-positive bacteria. Due to its extensive degree of modification, Pzn is highly protected from premature degradation by peptidases within the plant rhizosphere (Scholz et al., 2011). Remarkably, the human pathogen *B. anthracis* was found sensitive against PZN and underwent massive lysis at 4 μg mL^-1^ (Molohon et al., 2011). The exact structures of plantazolicin A and B, were elucidated, unveiling a hitherto unusual number of thiazoles and oxazoles formed from a linear 14mer precursor peptide (Kalyon et al., 2011).

Parasitic nematodes of plants are important plant pathogens that represent a significant financial burden on agriculture. FZB42 has been shown to reduce nematode eggs in roots, juvenile worms in soil, and plant galls on tomato (Burkett-Cadena et al., 2008). In order to identify specific-nematicide-related genes, a random transposon insertion library of FZB42 was screened for relevant genes involved in nematicidal activity and – surprisingly – a gene within the *pzn* gene cluster was identified as a pathogenic factor against nematodes. Further experiments revealed that PZN displayed a moderate nematicidal activity (Liu et al., 2013).

**FIGURE 3:**
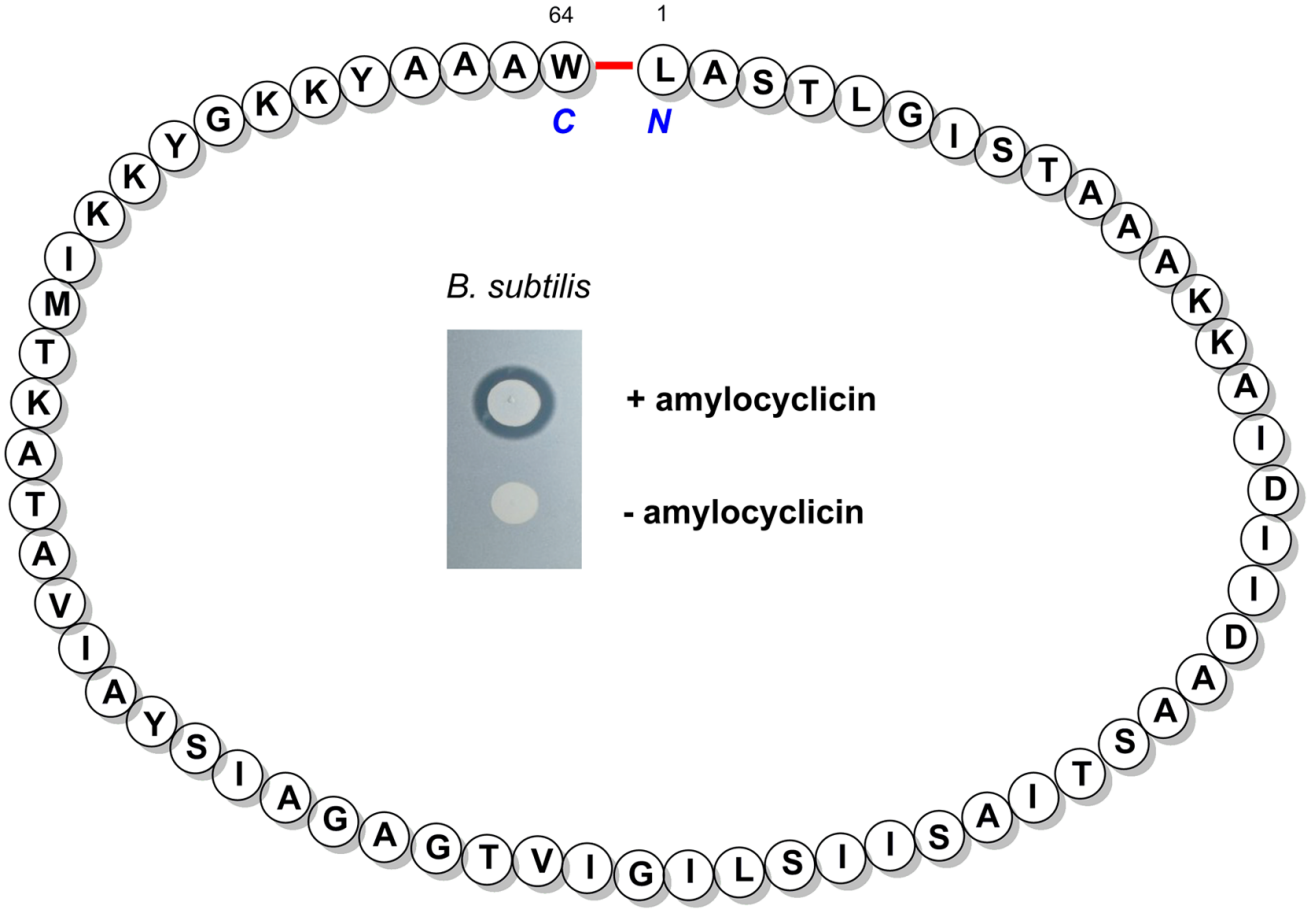
**The structure of the mature bacteriocin amylocyclicin bearing a head-to-tail cyclization of L_1_ and W_64_.** Inset: Amylocyclicin effect on a related *B. subtilis* strain without immunity against the bacteriocin was demonstrated by a spot-on-lawn test performed with a amylocyclicin producing **(Top)** and non-producing FZB42 strain **(Bottom)**.

By transposon mutagenesis of the FZB42 mutant strain RS06, which is deficient in Sfp-dependent synthesis of LP, polyketides, and bacilysin, we identified a hitherto unknown gene cluster involved in synthesis and posttranslational processing of a novel circular bacteriocin, named amylocyclicin (**Figure 3**). It became apparent that amylocyclicin inhibits growth of bacterial strains closely related to FZB42 suggesting that this bacteriocin might have a function in competing with other *Bacillus* strains attracted to the plant rhizosphere (Scholz et al., 2014).

## Persistence of FZB42 and its Impact on the Root Microbiome

It is commonly accepted, that the structure of the microbial community colonizing plant roots is important for the plant’s well-being and its resistance against pathogens. The root microbiota is strongly affected by soil type, as well as by the genotype of the host plant (Hartmann et al., 2009; Bulgarelli et al., 2012). FZB42 is able to colonize roots of lettuce plants growing in sandy loam soil, but presence of vegetative cells at the root surface becomes slowly reduced around 6 weeks after inoculation suggesting, that FZB42 is not a strong competitor of the indigenous root microflora (Borriss, 2015). However, our results demonstrated that FZB42 is able to reduce the disease severity of bottom rot caused by soil-borne pathogen *R. solani* on lettuce (Chowdhury et al., 2013).

As revealed by T-RFLP community fingerprinting and taxonomic profiling of metagenome sequences, application of FZB42 on field grown lettuce, independent of its mode of application, did not shift the composition of rhizosphere bacterial community in a measurable extent (Chowdhury et al., 2013; Kröber et al., 2014). Similar results were also found for *B. amyloliquefaciens* BNM122 in soybean (Correa et al., 2009). By contrast, inoculation with the pathogen did change the rhizosphere microbial community structure. Using the same setup in greenhouse experiments, the effect of FZB42 and the pathogen *R. solani* on the microbial community of lettuce was more deeply analyzed by 454-amplicon sequencing focusing on the presence of gamma-proteobacteria (Erlacher et al., 2014). Clear differences between plants infected by *R. solani* compared to non-inoculated healthy plants were found, corroborating the results obtained by T-RFLP. A significant increase in gamma-proteobacterial diversity was detected in samples inoculated with the pathogen, whilst in the presence of FZB42 and the pathogen together this increase was less distinct, suggesting a selective compensation of the impact of a pathogen on the indigenous plant-associated microbiome by FZB42. The method called ‘fragment recruitments’ was used to track the persistence of the FZB42 inoculant. The number of DNA fragments corresponding to FZB42 decreased in the course of the plant cultivation. After 5 weeks, about 55% of the initial number of FZB42 DNA was still traceable within the rhizosphere of lettuce in the field (Kröber et al., 2014).

## Plant Defense is Triggered by Plant-Associated Bacilli including FZB42

The biocontrol effect shown by FZB42 could rely on the potential antimicrobial activity of several bioactive secondary metabolites. However, except surfactin, concentration of antifungal LP determined *in planta* was found relatively low (Nihorimbere et al., 2012; Chowdhury et al., 2015). Moreover, antibacterial polyketides and other bioactive compounds were not detected so far in the vicinity of plant roots colonized by PGPR Bacilli (Debois et al., 2014). Therefore, it is tempting to speculate that ISR triggered by surfactin, microbial volatile organic compounds (mVOCs) and, possibly, other hitherto unidentified secondary metabolites, is a main factor in suppressing plant pathogens by PGPR Bacilli. ISR is defined as ‘enhanced defensive capacity of the entire plant against a broad spectrum of pathogens; acquired upon local induction, e.g., at roots, by beneficial microbes’ (Pieterse et al., 2014). ISR was initially demonstrated in *Pseudomonas* sp. and other Gram-negative root associated bacteria, but later it was demonstrated that several *Bacillus* sp. including *B. subtilis* and *B. amyloliquefaciens* elicit ISR in *Arabidopsis*, several vegetables, tobacco, and tropical crops (Kloepper et al., 2004). Typically, the rhizobacteria induce plant defense via jasmonic acid (JA) and/or ethylene (ET) signaling pathways. ISR is distinct from systemic acquired resistance (SAR) in which the response is triggered by pathogenic microorganisms associated with the aerial portions of the plant. In systemic tissues, SAR is characterized by increased levels of the hormone salicylic acid (SA). Interestingly, plants treated with *B. pumilus* had greatly increased levels of SA, compared to the untreated control (Zhang et al., 2002).

**FIGURE 4:**
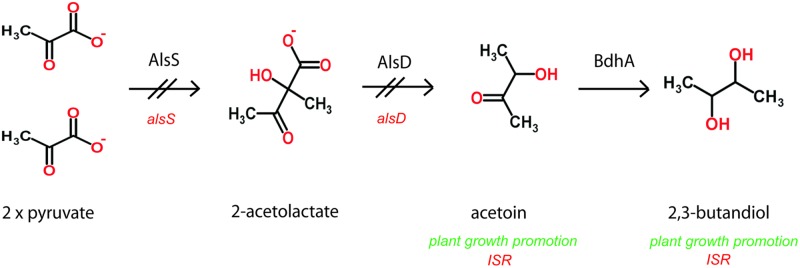
**Anaerobic and aerobic formation of 2,3-butanediol *via* acetoin involves acetolactate synthase and decarboxylase encoded by the *alsSD* operon.** The *alsS* insertion mutation abolishes synthesis of 2,3-butandiol (Renna et al., 1993; Cruz Ramos et al., 2000).

Selected *Bacillus* PGPR strains emit mVOCs that can elicit plant defenses. Exposure to VOCs consisting of 2,3-butanediol and acetoin (3-hydroxy-2-butanone) from PGPR *B. amyloliquefaciens* activates ISR in *Arabidopsis* seedlings (Ryu et al., 2003). *Arabidopsis thaliana* plants exposed to *B. subtilis* strain FB17, results in reduced disease severity against *Pseudomonas syringae* compared to plants without FB17 treatment. Exogenous application of acetoin triggers ISR and protects plants against the pathogen in the aerial parts (Rudrappa et al., 2010). In this context it is worth to mention, that expression of acetolactate synthase, AlsS of FZB42, an enzyme involved in the synthesis of acetoin (**Figure 4**) was strongly increased in the presence of maize root exudates during late exponential growth phase (Kierul et al., 2015), suggesting that root exudates play a role in eliciting of acetoin biosynthesis in FZB42. In the same study, it was found that two proteins related to chemotaxis and motility, flagellar hook-associated protein II (FliD) and the Hag flagellin protein, were increased in the presence of root exudates. Flagellin proteins bearing the flg22 elicitor signal are recognized by most plant plasma membrane-localized pattern recognition receptors and are thus thought to prime plant basal defense against potential pathogens. *B. amyloliquefaciens* FZB24 and FZB42 applied to tobacco roots led to a reduction of tobacco mosaic virus symptoms visible on tobacco leaves, and to decreasing amounts of virus proteins present in leaf tissues. Due to the spatial distance between the beneficial bacterium and the pathogen, plant ISR, stimulated by the rhizobacterium, might be responsible for this effect. In fact, it was shown that the application of PGPR Bacilli led to ISR toward viral infection and enhanced plant growth (Wang et al., 2009).

The induction of ISR when treated with Gram-negative PGPRs is mediated primarily through the plant hormones JA, a lipoxygenase pathway product, and ET. By contrast, SA appears to be a critical plant messenger of pathogen exposure and disease resistance in SAR (Pieterse et al., 2014). Notably, a simultaneous activation of ET and SA-signaling pathways by *Bacillus* sp. was found when defense compromised mutants of *Arabidopsis thaliana* were exposed to FB17, whilst JA was not essential (Rudrappa et al., 2010).

In order to determine the signaling pathways triggered by FZB42, the expression of several marker genes in lettuce plants, exposed to FZB42 and the pathogenic fungus *R. solani*, were analyzed by quantitative real time (RT)-PCR (Chowdhury et al., 2015). In absence of the pathogen, FZB42 increased expression of *PR1* (pathogenesis protein 1, SA marker gene), and plant defensing factor 1.2 (*PDF1.2*; defensin, JA/ET marker gene), suggesting that SA and ET pathways are involved in up-regulating defense response in lettuce. In simultaneous presence of FZB42 and the pathogen *R. solani, PDF1.2* expression was dramatically enhanced, suggesting a synergistic activation of the JA/ET pathway, whilst the SA pathway – as indicated by a decreased expression of *PR-1* – was suppressed in presence of both antagonists. This is in accordance with previous results (Sarosh et al., 2009) suggesting that *B. amyloliquefaciens* can induce systemic resistance in oilseed rape against *Botrytis cinerea* through a JA dependent *PDF 1.2* expression.

The circular LP surfactin and to a minor extent fengycin can act as elicitors of host plant immunity and contribute to increased resistance toward further pathogenesis development in bean and tomato plants (Raaijmakers et al., 2010). In bean, purified fengycins and surfactins provided a significant ISR-mediated protective effect against the fungal pathogen *Botrytis cinerea*, similar to the one induced by living cells of the producing strain *B. amyloliquefaciens* S499 (Ongena et al., 2007). Similarly, low concentrations of surfactin induced several early plant-defense related events in tobacco cells (Jourdan et al., 2009). A strong correlation was found between defense-inducing activity (e.g., stimulation of ROS production) and the amount of surfactin produced by several isolates belonging to the *B. subtilis*/*amyloliquefaciens* complex (Cawoy et al., 2014). Combined application of surfactin and live cells of mutant strain FZB42-AK3 (produces surfactin, but not bacillomycin D and fengycin) reduced gray leaf spot disease caused by *Magnaporthe oryzae* in ryegrass *Lolium perenne*. A multilayered ISR defense response in ryegrass cells was registered, such as: (1) enhanced accumulation of hydrogen peroxide (H_2_O_2_), (2) elevated cell wall/apoplastic peroxidase activity, (3) deposition of callose and phenolic/polyphenolic compounds underneath the fungal appressoria in leaves, (4) hypersensitive response (HR)-type reaction together with enhanced expression of peroxidase, oxalate oxidase, phenylalanine ammonia lyase, lipoxygenase, and putative defensins (Rahman et al., 2014).

We found that the dramatic increase of the *PDF1.2* gene expression in simultaneous presence of *B. amyloliquefaciens* and *R. solani* occurred only when wild type cells of FZB42 were applied. Mutant strains deficient in non-ribosomal synthesis of LP and polyketides did not stimulate expression of the JA/ET pathway, suggesting that cyclic LP contribute to the ISR plant response triggered by FZB42 (Chowdhury et al., 2015).

## Conclusion

Biocontrol of plant pathogens is an important feature of *Bacillus* inoculants applied for a more sustainable agriculture. FZB42, when added to plants, does not affect the root microbiome, but FZB42 seems to restore, at least in part, the original community structure which has been previously altered by competing plant pathogens, such as fungus *R. solani*.

**FIGURE 5:**
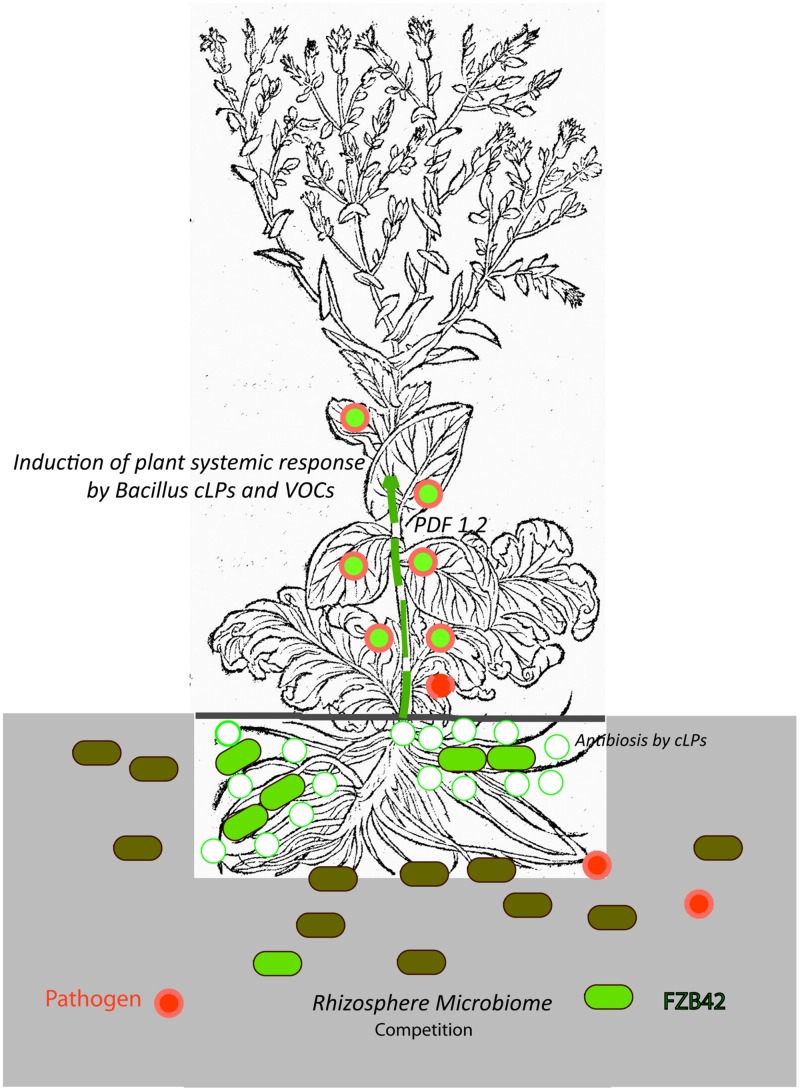
**Biological control exerted by the plant-beneficial bacterium FZB42.** The cartoon illustrates our present picture about the complex interactions between a beneficial Gram-positive bacterium (FZB42, light green), a plant pathogen (*R. solani*, symbolized by red filled circles) and plant (lettuce, *Lactuca sativa*). FZB42 colonizes the root surface and is able to produce non-ribosomally cyclic lipopeptides, mainly surfactin and bacillomycin D and to a minor extent fengycin as indicated by the green circles (Chowdhury et al., 2015). It is very likely, but not shown until now, that VOCs (e.g., acetoin, 2,3-butandiol), and small peptides (e.g., plantazolicin, amylocyclicin) are also produced in vicinity of plant roots. Direct antibiosis and competition for nutrients (e.g., iron) suppresses growth of bacterial and fungal plant pathogens in the rhizosphere. However, these effects seem to be of minor importance, since the composition of the root microbiome is not markedly affected by inoculation with FZB42 (Erlacher et al., 2014), and the number of vegetative *B. amyloliquefaciens* cells on root surfaces is steadily decreasing (Kröber et al., 2014). Due to production of *Bacillus* signaling molecules (cLPs and VOCs) and in simultaneous presence of *R. solani*, the plant defensing factor 1.2 (*PDF1.2*) as indicated by the green-filled red circles is dramatically enhanced and mediates defense response against plant pathogens (Chowdhury et al., 2015). The picture of the *lettuce* plant (*Lactuca crispa*) was taken from Bock, 1552, p. 258).

Recent results mainly obtained with *B. amyloliquefaciens* FZB42 and other representatives of the *B. amyloliquefaciens plantarum* subspecies support the hypothesis that stimulation of plant ISR by bacterial metabolites, such as surfactin and volatiles, is the key mechanism in the biocontrol action of Gram-positive endospore-forming bacteria. By contrast, a direct effect of the numerous antimicrobial secondary metabolites in suppressing pathogens occurring in the plant rhizosphere seems to be of minor importance. In addition, sublethal concentration of other LP, such as fengycin and iturins, might prime plant defense response against plant pathogens. The role of non-ribosomal polyketides and of an emerging number of small peptide molecules (bacteriocins) ribosomally synthesized by FZB42 and other PGPR Bacilli in suppressing concomitant phytopathogens occurring in plant rhizosphere remains elusive. **Figure 5** summarizes the main points of our present knowledge about the effect of plant-associated *Bacillus* within the tripartite system consisting of beneficial Gram-positive bacterium, pathogen and plant.

Future work will focus on further elucidating the effects exerted by secondary metabolites produced by plant-growth-promoting Bacilli within plant rhizosphere on plant health and growth. The response of the plant in simultaneous presence of the beneficial *Bacillus* and the pathogen will be of special interest. In this context, use of a model bacterium such as FZB42 seems to be advantageous and should make results more comparable.

## Conflict of Interest Statement

The authors declare that the research was conducted in the absence of any commercial or financial relationships that could be construed as a potential conflict of interest.
